# Conservation, Spillover and Gene Flow within a Network of Northern European Marine Protected Areas

**DOI:** 10.1371/journal.pone.0073388

**Published:** 2013-09-06

**Authors:** Mats Brockstedt Olsen Huserbråten, Even Moland, Halvor Knutsen, Esben Moland Olsen, Carl André, Nils Chr. Stenseth

**Affiliations:** 1 Centre for Ecological and Evolutionary Synthesis (CEES), Department of Biosciences, University of Oslo, Oslo, Norway; 2 Institute of Marine Research (IMR), Arendal, Norway; 3 Department of Natural Sciences, University of Agder, Kristiansand, Norway; 4 Department of Biology and Environmental Sciences-Tjärnö, University of Gothenburg, Strömstad, Sweden; Bangor University, United Kingdom

## Abstract

To ensure that marine protected areas (MPAs) benefit conservation and fisheries, the effectiveness of MPA designs has to be evaluated in field studies. Using an interdisciplinary approach, we empirically assessed the design of a network of northern MPAs where fishing for European lobster ( *Homarusgammarus* ) is prohibited. First, we demonstrate a high level of residency and survival (50%) for almost a year (363 days) within MPAs, despite small MPA sizes (0.5-1 km^2^). Second, we demonstrate limited export (4.7%) of lobsters tagged within MPAs (N = 1810) to neighbouring fished areas, over a median distance of 1.6 km out to maximum 21 km away from MPA centres. In comparison, median movement distance of lobsters recaptured within MPAs was 164 m, and recapture rate was high (40%). Third, we demonstrate a high level of gene flow within the study region, with an estimated *F*_ST_ of less than 0.0001 over a ≈ 400 km coastline. Thus, the restricted movement of older life stages, combined with a high level of gene flow suggests that connectivity is primarily driven by larval drift. Larval export from the MPAs can most likely affect areas far beyond their borders. Our findings are of high importance for the design of MPA networks for sedentary species with pelagic early life stages.

## Introduction

Marine protected areas (MPAs) and marine reserves are increasingly recognised as tools to rebuild marine ecosystems and fisheries suffering from overexploitation [1,2]. Typically, marine reserves display a marked increase in biomass, density, size, and diversity of species that are harvested outside the reserves [3,4]. From a fisheries perspective, a measurable effect of a marine reserve is net export of adults and juveniles from reserves and into adjacent, fished areas (spillover) [5]. Furthermore, protecting large, highly fecund individuals within reserves may lead to increased export of pelagic eggs and larvae (recruitment benefits) [6]. To ensure both conservation and fisheries benefits of reserves, there should be a balance between protection and spillover–a balance regulated by reserve design [7]. At the same time, where larvae exported from reserves end up, is unknown for most species of commercial interest [1]. However, measuring gene flow by genetic markers can elucidate both magnitude and distance of effective larval dispersal [8–10]. Estimates of larval dispersal distances can subsequently be used to optimise reserve placing within a network, ensuring connectivity among reserves [11], which in turn increases sustainability of reserves [12].

**Figure 1:**
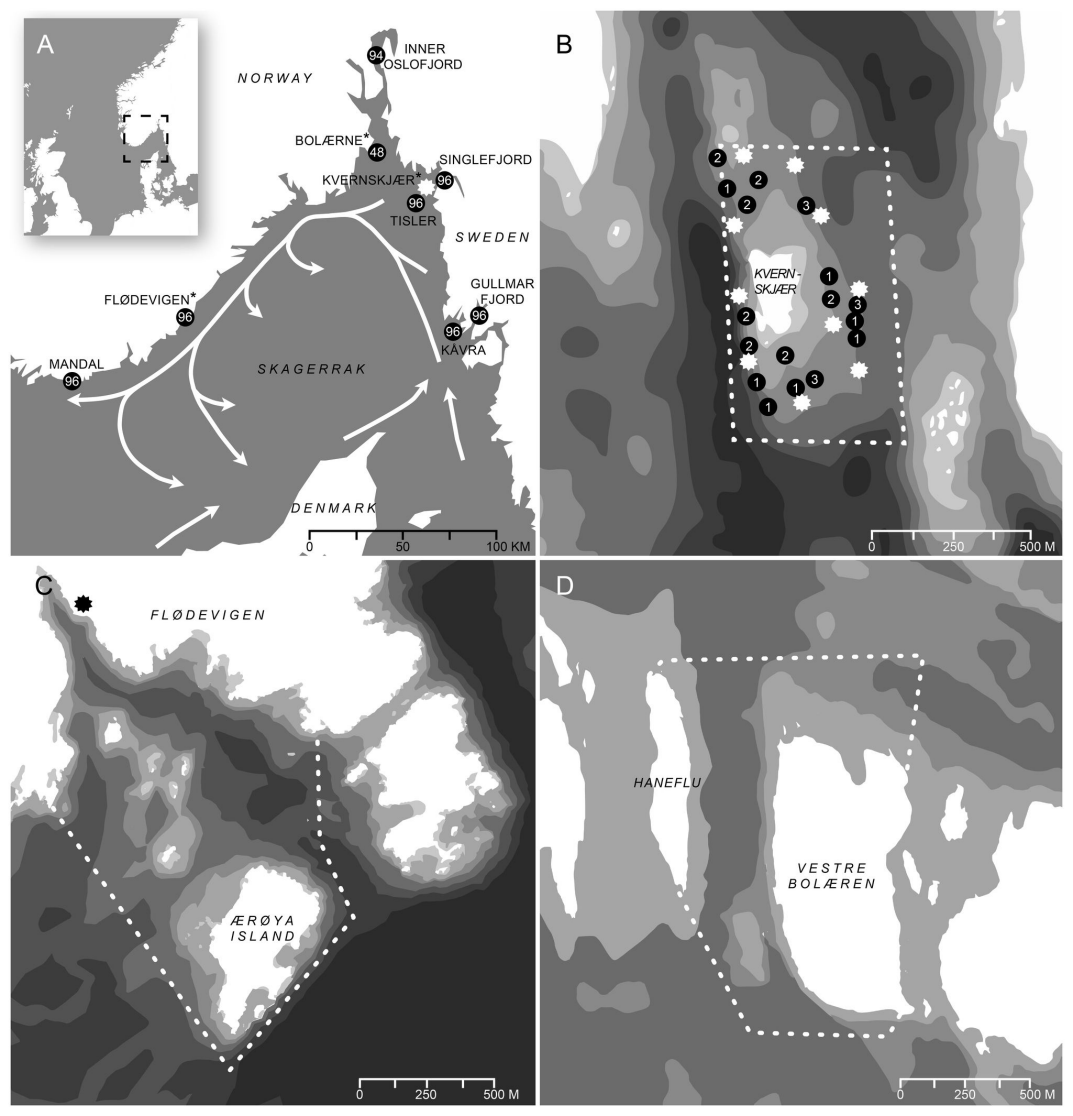
Illustrations of study areas in Skagerrak. (A) Illustration of Skagerrak, depicting samples for microsatellite DNA markers (black circles) and telemetry study site (white star). Sites with asterisk (*) indicate lobster reserves monitored during scientific fishing programme. White arrows represent large-scale water movement in Skagerrak, redrawn from [22]. (B) Map of Kvernskjær lobster reserve, delineating border of lobster reserve (white dotted line), VR2W receiver array (white stars), and release sites of lobsters fitted with acoustic transmitters (black circles). (C) Map of Bolærne lobster reserve. (D) Map of Flødevigen lobster reserve, where black star indicates the Flødevigen research station. Every change in grey in bathymetric maps represents a 10 m isobath (except the lightest, which includes the depth range of 1 m-0 m).

To ensure that MPAs and marine reserves provide their proposed benefits to conservation and fisheries, the effectiveness of current reserve design has to be evaluated in field studies [12]. Here we assess the design of a network of northern MPAs within which fishing for the European lobster (Homarus gammarus) is not allowed (hereafter termed lobster reserves or reserves). This network consists of three reserves, situated along the Norwegian Skagerrak coast (Figure 1A). The reserves were established in 2006, and already in 2010 abundances of lobsters within the reserves had more than tripled and mean size had increased significantly [13]. This in contrast to a precarious fishery in Skagerrak were official catch-per-unit-effort (CPUE) data provided by fishers operating along the Norwegian Skagerrak coast shows a clear decline since the early to mid 1900s (Figure S1); a decline correlated with the ever-increasing fishing pressure exerted by recreational participants in the popular fishery [14]. CPUE of lobsters has also drastically decreased along the Swedish part of the Skagerrak coast in recent years (1950-2010) compared to earlier years (1875-1950); and in addition, during this period (1875-2010) the lobster population have changed from a naturally regulated state, characterised by periodic fluctuations, into a heavily exploited fisheries controlled stock with less variability [15]. Whereas catches in Scotland, England and Wales, and France have been variable, yet without clear trends from 1930 to 1997 [16]. Current management regulations in Norway include a maximum of 10 and 100 traps per recreational and commercial fisher; a minimum landing size (MLS) of 25 cm total length (≈ 90 mm carapace length, CL); and a trade and landings ban on ovigerous females. Otherwise, lobsters are legally harvested when equal to or greater than MLS in traps fitted with two circular escape vents measuring 60 mm during a two-month fishing season (1 October to 30 November).

Besides a lack of empirical knowledge on the connectivity of the European lobster, its general biology is fairly well known. Although the female lobster reproduction cycle is subject to variation, it is generally considered to last two years; they moult and mate the first summer, and extrude their eggs the following summer. Eggs hatch the next summer, after which the females immediately moult and mate [17]. Females carry their eggs from 9 to 11 months, and hatching usually occurs from April to July [18]. The four subsequent pelagic larval stages are predominantly found in the neuston, while they display a strong diel vertical migration [19]. Settlement follows 13 to 35 days in the pelagic zone [18].

To assess the effectiveness of the Skagerrak lobster reserve network we performed a three part study, with the aims to test: (1) to what degree reserves offer long-term protection of lobsters, (2) the level of spillover from reserves to adjacent fisheries, and (3) the level of gene flow among areas harbouring lobster reserves. To address these aims, we collected data at three spatial levels along coastal Skagerrak (Figure 1A). First, to determine if reserves protect their inhabitants over extended time periods, we monitored the fates of 30 lobsters within the Kvernskjær reserve by acoustic telemetry over a year. Second, to determine if reserves provide spillover, and if so the magnitude and extent of spillover, we collected capture-mark-recapture and recovery data within and around the Kvernskjær, Flødevigen, and Bolærne lobster reserves (Figure 1B, C, D) over five years (2006-2010). Third, to elucidate the connectivity of lobsters in Skagerrak, and thus the potential for reserves to seed the fished stock and other reserves, we quantified the genetic heterogeneity among lobster aggregations at eight localities along the entire Skagerrak coast, where three of these aggregations were sampled within reserves (Figure 1A). The eight genetic sampling localities were more or less exposed to the Norwegian coastal water (NCW) current, moving counterclockwise along the Skagerrak coast at mean speeds of 10-40 cm^-s^ during summer, weakly dissipating in deeper layers. For example, upper water masses may take approximately two weeks to move along the entire Skagerrak coast [20]. Skagerrak surface water is also influenced by wind conditions that may be highly variable in the ‘dispersal window’ of lobster larvae. To the best of our knowledge, no study has so far evaluated the design of a coastal network of MPAs by integrating information on multiple spatial scales (from fine-scale movement within reserves to long-distance dispersal among sub-populations). Our results could therefore be of major importance for the future design of MPA networks for sedentary species with pelagic early life stages.

## Materials and Methods

### Study sites

The acoustic telemetry study site, the Kvernskjær lobster reserve (0.5 km^2^, Figure 1B), is situated around a small islet within the Hvaler archipelago on the eastern side of the Oslofjord’s outlet. SCUBA surveys within the reserve revealed that macro-algae were sparse near the surface, due to strong fresh water influence from the river Glomma. However, macro-algae are present from 5 m-12 m, and the submerged plateau at the southern end of Kvernskjær Island contains a sparse kelp forest. Steep slopes consist of rock faces with boulders at their base. Kvernskjær Island is flanked by a particularly steep slope and deep channel (> 50 m) on the western side. In deeper basins and flat areas the bottom consists of sand and mud.

The Flødevigen lobster reserve (1 km^2^, Figure 1C) contains a main gully that gains depth as it cuts from northwest to southeast, emptying into the deeper basin north of Ærøya Island. Depths increase gradually and range from 0 to < 50 m. In deeper basins and flat areas the bottom consists of shell sand, sand, or mud. The greatest depths are found on the reserve boundary perpendicular to the steep slope on the exposed side of Ærøya Island. Most of this reserve is protected from the Skagerrak Sea by an archipelago. The upper 1-5 m of substrate within the Flødevigen reserve is mostly rocky habitat dominated by macro-algae. Photosynthetic communities become gradually sparser down to 10 m and are more or less absent at greater depths, except for the exposed south-eastern side of Ærøya Island, which contains a lush kelp forest that extends down to 10-12 m.

The Bolærne lobster reserve (0.7 km^2^, Figure 1D) is situated on the western side of the Oslofjord. The reserve harbours relatively shallow (< 20 m) hard bottom habitat with sparse macro-algae. However, the channel between the two islands Haneflu and Vestre Bolæren is deeper than 20 m in some areas with sediments mainly consisting of shell sand. Some larger rocks and cobble lies at the slope bottom on each side of this channel. The topography of the coastline along which the three lobster reserves are situated was created by glacial scouring, harbouring typical lobster habitat representative of coastal Skagerrak.

### Acoustic telemetry

To estimate survival and residency of lobsters within the Kvernskjær reserve over a year, we fitted a Kaplan-Meier survival curve [21] to the data described below. Based on telemetric signals containing depth readings from 30 lobsters, we categorised each individual’s status daily as: present and moving (i.e. alive) within the reserve, lost from the study, or censored. Individuals interpreted as alive within the reserve displayed a diel vertical movement considered ‘normal.’ Cessation of signals with unknown cause placed individuals in the category ‘lost’. This could for example mean movement out of the listening range of receivers or tag loss due to moulting (e.g., tag lying under rocks or sinking into soft substratum). Nevertheless, these individuals were no longer in the study. Censored individuals typically emitted depth readings 2 m above, and 110 m below sea level within short time intervals, physically impossible within our study area. This was interpreted as a malfunctioning transmitter, and after such an event its signal was not considered trustworthy. An important assumption of the censored individuals is that their ‘survival’ time is at least longer than the time of their censoring. The rationale behind the Kaplan-Meier survival curve (hereby referred to as a loss curve, with respective loss rates over given time intervals) is that it quantifies the probability of a lobster being alive within the listening range of the receivers until a given time, explicitly taking into account the censored individuals [21]. For the number of days each telemetry tagged individual was present in the study, along with their categorised ‘fate’, see supporting information (Table S1). The 30 lobsters monitored consisted of a sample of 10 females, 10 males, and 10 ovigerous females. The 30 individuals were classified into one of four successive moult stages [22] before the study (Table S1). Eggs of all ovigerous females were in development stage one, of four possible stages [23]. Hence, ovigerous females would not moult until the following year, subsequent to the hatching of their eggs. To determine if ovigerous females, males, and females had equal probabilities of being protected by the reserve, and if moult stage at start of study affected loss curves (i.e., if earlier moult stages were lost before later moult stages) the log-rank test was used. The lobsters were trapped at 10-30 m depth during 24 to 26 August 2008. Before their release, the lobsters were equipped with V13P-1H high power coded acoustic transmitters with depth sensors (Vemco Ltd., Halifax, Canada, diameter 13 mm, length 45 mm, weight in seawater: 6 g, emitting on 69 kHz). Tags were programmed to emit at a randomised interval between 110 and 250 s and had an expected battery life of around 500 days. Tag harnesses were constructed and fitted to the middle segment of the crusher claw [24]. Subsequently, lobsters were released at their capture locations within the Kvernskjær reserve (Figure 1B). Note that these lobsters would lose their acoustic transmitter when moulting. During the period 27 August 2008 to 24 August 2009, 10 VR2W receivers (Vemco Ltd, Halifax, Canada) were deployed to monitor the 30 lobsters fitted with acoustic transmitters (Figure 1B). VR2W receivers were moored to concrete anchors with a rope, and kept erect in the water column at 9 m below surface by a trawl float attached 1 m above the receiver. Except for shallow areas close to the Kvernskjær Island, the listening range of these receivers covered the entire reserve area. This was revealed by a range testing study where we lowered a range-testing transmitter (V16-4H, Vemco Ltd.) to the bottom at 70 sites throughout the reserve (Figure S2).

### Capture-mark-recapture and recovery

To quantify the magnitude of export from reserves, we depended on local fishers to report if they recovered any tagged lobsters outside the reserves during the two-month fishing season. To determine the spatial extent of the export from reserves, we used recovery locations provided by fishers to measure movement distances. Additionally, to quantify movement within the reserves, we measured the distance moved between capture- and subsequent recapture positions within the reserves. Movement distances inside reserves were measured as a straight line, from release site to the geographic location where the lobster was trapped. If a lobster was recaptured within a reserve multiple times, one movement distance was calculated for each time interval between recaptures. Movement distances outside the reserves were measured as a straight line from reserve centre to recovery location. To determine if movement distances from reserves was correlated with days since last capture within reserve (i.e., days at liberty), Kendall’s τ was used. We tagged lobsters within the Kvernskjær, Flødevigen, and Bolærne reserves annually (in August) since 2006. Every year, 100 trap days were set within each reserve over four days, each day with a 24 hour soak time (25 traps per day * four days = 100 trap days per reserve per year) [13]. Until and including 2010 (i.e. after ≈1500 trap days over five years), a total of 1810 lobsters had received T-bar anchor tags (TBA1, 30 x 2 mm, Hallprint Pty. LTD, Holden Hill, South Australia) with printed information about the scientific fishing programme. Tagged lobsters were always released at their trapping positions within the reserves. For details on how many lobsters were tagged within each reserve each year and their size distribution (separated by sex), see supporting information (Table S2 and Figure S3).

### Genetic markers

To test the level of genetic heterogeneity within Skagerrak, 718 individuals from eight samples (see Figure 1A for sample size at each locality) were screened for genetic variability using 12 microsatellite DNA markers. Lobsters sampled in Flødevigen, Bolærne, and Kåvra where caught within reserves, whereas individuals from the other sites where obtained from fishers in the given area. DNA of individual lobsters was extracted from muscle tissue of one of their pleopods, and microsatellite loci were amplified using published PCR conditions [25]. Deviations from Hardy-Weinberg (HW) equilibrium, for geographic samples and each locus, were quantified using the *F*_IS_ statistic, and to test for excess or deficiencies of heterozygotes, we used the two-sided HW probability tests. Both the HW disequilibrium estimation (*F*_IS_) and probability test were performed in GENEPOP [26]. Linkage disequilibrium and presence of null alleles was ruled out using GDA [27] and MICRO-CHECKER [28]. We also tested that none of the microsatellite loci were affected by either directional or balancing selection (Figure S4), using LOSITAN [29,30]. Further, we calculated Weir & Cockerham’s F_ST_ estimator θ [31] from allele frequencies both overall samples and between sample pairs using GDA. Confidence intervals around F_ST_ for each locus, and for the overall F_ST_, were estimated by jack-knifing over populations and by bootstrapping over loci. To test for isolation by distance, the pairwise distance (km) between sampling locations was regressed on pairwise F_ST_. Here an increasing trend in F_ST_ would be a clear sign of restricted dispersal of lobsters within our study region, and a lack of trend would suggest little restriction to dispersal [9]. Allele frequency tests of differentiation were performed in GENEPOP and we used False Discovery Rate (FDR) correction for multiple testing [32].

### Ethics statement

The necessary permissions for capture-release, tagging, and collection of lobster tissue samples were obtained from the Norwegian Animal Research Authority. The Norwegian Directorate of Fisheries provided the necessary permission to conduct sampling/ experimental fishing outside of the lobster fishing season and inside MPAs. Although the European lobster is not considered endangered, it figures as “near threatened” in the Norwegian Red List according to IUCN (International Union for Conservation of Nature) criteria [33], but has no such status in the Swedish Red List. In both countries the species is subject to a managed fishery. Norwegian and Swedish tissue samples collected away from MPAs and control areas monitored by the Institute of Marine Research were provided by participants in the lobster fishery, collected during the lobster fishing season. For this reason, no permission was required for collection of tissue samples from these sites.

## Results

### Survival and residency within a lobster reserve

**Figure 2:**
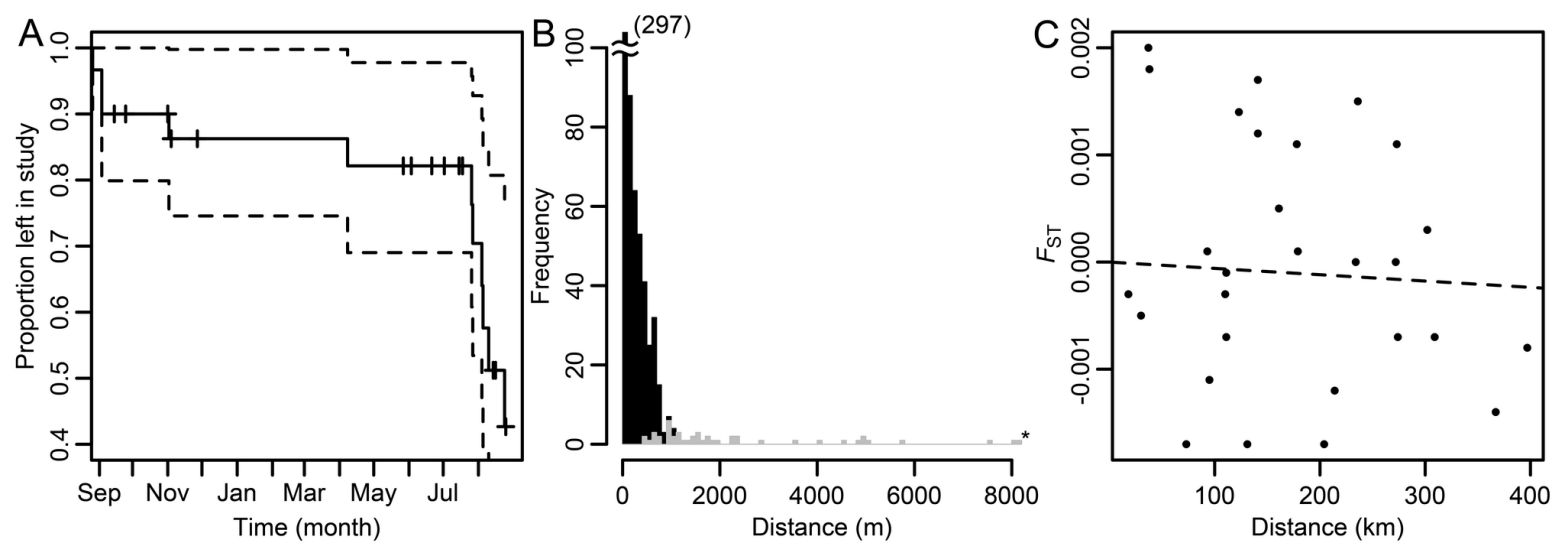
Survival and residency, spillover, and gene flow of lobsters in Skagerrak. (A) Survival and residency of 30 lobsters within a MPA over one year. Crosses indicate censored individuals, where the depth sensor has emitted nonsensical depth readings and the individual is out of the study. (B) Movement distances by lobsters tagged within reserves and recovered by recreational and commercial fishers outside reserves (grey bars), and recaptured by the authors within the reserves during the annual research trapping (black bars). The bars are stacked and movement distances are classified into increments of 100 m. *Note that four lobsters were recovered by fishers beyond 8200 m (11.5, 15, 15, and 21 km away from reserve centres). (C) Regression between pairwise F_ST_ and geographic distance between samples.

The median number of days (i.e., the time until survival probability was 0.5) the lobsters monitored by acoustic telemetry were alive and moving within the Kvernskjær reserve was 363 days. From November to May few lobsters were lost from the study, while the loss rate was high from July to August (Figure 2A). There were no significant differences in loss curves among males, females, and ovigerous females. Neither did moult stage at start of study affect loss curves significantly. For further details on number of lobsters at risk over time (i.e. number of lobsters present and moving within the reserves until a given time) see supporting information (Table S3).

### Movement of lobsters within and from reserves

Of 1810 lobsters tagged and released within three lobster reserves, 85 (4.7%) were recovered by fishers outside the reserves. Out of these, 51 reports included size of lobsters at capture and geographical information on capture position (Table 1). Among the three reserves, the mean recovery percentage outside reserves was 4.5% (95% CI: 2.7-6.2%). In comparison, total recapture percentage within reserves was 40% and mean recapture percentage within the three reserves was 41% (95% CI: 2-80%). The median distance moved by all lobsters recovered outside reserves was 1.6 km and ranged from 0.5 to 21 km. The median distance moved within reserves, among 735 time intervals lasting at least one year, was 164 m and ranged from 2 to 1088 m (Figure 2B). Number of days lobsters had been at liberty was not significantly correlated with movement distance from reserve centre. For size distribution of lobsters recovered by fishers, see supporting information (Figure S5).

**Table 1:** Capture-mark-recapture and recovery data from three lobster reserves.

	Within			Outside		
Reserve	Tagged	Recaptured	Median movement distance (m)	Recovered	Median movement distance (m)	Mean days at liberty
Kvernskjær	527	335	358	20	2300	742
Flødevigen	531	131	59	23	1100	305
Bolærne	752	268	89	42	2150	381
Total	1810	735	164	85	1600	418

Data collected throughout five years of scientific monitoring (research fishing), including: number of individuals tagged within reserves; number of individuals recaptured within reserves and the median movement distance since last capture; number of lobsters recovered by fishers outside reserves and their median movement distance (if geographic position on recovery site was reported), and mean number of days recovered individuals had been at liberty since last capture within reserve.

### Gene flow

Of the eight localities sampled for microsatellite markers, three showed a deviation from HW equilibrium expectations after FDR corrections (Table S4). Though, within each of these three sub-populations, only two loci showed significant p-values at most. The proportion of genetic variation that could be partitioned among geographic samples (F_ST_) estimated for each locus was very low and ranged from 0.00227 to -0.00283 (Table 2). The mean F_ST_ across all loci was 0.00005, with 95% confidence limits of 0.00088 to -0.00080. Despite this very low level of F_ST_, allele frequencies varied significantly among geographic samples (p = 0.006, cf. Table 2), largely due to locus HGC131 (p = 0.006) that also had a high deficiency in heterozygotes compared to HW expectations (i.e. a high, positive *F*_IS_). The overall p-value without this locus was 0.019. There was no significant trend in pairwise F_ST_ (i.e. no isolation by distance) over the scope of our sampling area (Figure 2C). There were no significant differences in allele frequencies among pairwise comparisons of the geographic samples after FDR corrections (Table S5).

**Table 2:** Descriptive statistics of microsatellite data.

Locus (GenBank #)	Alleles	*H*_T_	*F*_ST_	Upper 95% CI	Lower 95% CI	P-value	FDR corr.
HGA8 (GU233660)	14	0.81	0.00085	0.00314	-0.00145	0.043	0.216
HGB4 (GU233661)	7	0.64	-0.00283	-0.00116	-0.00449	0.971	0.971
HGB6 (GU233662)	11	0.79	-0.00130	-0.00005	-0.00256	0.395	0.526
HGC6 (GU233663)	10	0.38	0.00140	0.00434	-0.00154	0.115	0.276
HGC103 (GU233664)	9	0.69	-0.00036	0.00281	-0.00354	0.518	0.565
HGC111 (GU233665)	14	0.76	0.00045	0.00365	-0.00275	0.242	0.414
HGC118 (GU233666)	10	0.55	-0.00002	0.00361	-0.00365	0.074	0.222
HGC120 (GU233667)	18	0.86	0.00023	0.00283	-0.00238	0.192	0.384
HGC129 (GU233668)	10	0.59	-0.00072	0.00119	-0.00263	0.054	0.216
HGC131 (GU233669)	23	0.85	0.00218	0.00438	-0.00002	0.006	0.072
HGD106 (GU233670)	11	0.69	-0.00178	0.00009	-0.00366	0.503	0.565
HGD111 (GU233671)	14	0.60	0.00227	0.00716	-0.00262	0.299	0.448
Overall			0.00005	0.00088	-0.00080	0.006	

Name of locus (with GenBank access number), number of alleles and average heterozygosity at locus (*H*_T_); proportion of genetic variation partitioned among geographic samples (F_ST_), with corresponding 95% confidence limits; p-values from allele frequency tests; and FDR corrected p-values.

## Discussion

Through a multidisciplinary empirical approach, this study aimed to quantify the level of protection, spillover, and gene flow of a harvested species, the European lobster, within a network of MPAs. The probability of long-term residency and survival within a reserve was 0.5 until approximately one year (363 days), and the percentage of lobsters tagged inside reserves, caught outside in the fishery was 4.7%. Gene flow among coastal sub-populations was high, as indicated by the low overall F_ST_. These findings, and their potential implications for management, are further discussed below.

### Skagerrak lobster reserve sizes matches the lobster’s scale of movement

To ensure that marine reserves protect their inhabitants, there should be a correlation between reserve size and their inhabitant’s scale of movement [7,11]. Lobsters monitored by acoustic telemetry had a 50% probability of being present and moving, i.e. of being protected per se, within the Kvernskjær reserve for nearly a year (363 days). This is also consistent with the high average recapture percentage of T-bar tagged lobsters within the reserves (41%) and short movement distances within reserves (median = 164 m). Moreover, 95% of lobsters tracked within the Flødevigen reserve have been shown to reside within, or near the reserve’s boundaries for up to one year [24]. To conclude, despite the small sizes of the Skagerrak lobster reserves, they appear to match the European lobster’s scale of movement. Consequently, the reserves reduce fishing mortality sufficiently to allow density to increase and size structure to expand within reserves compared to outside [13,34], prerequisites for the recruitment benefit. However, although per-capita and per-unit-area egg production of lobsters undoubtedly increases within Skagerrak reserves due to increased density and size of the lobsters [35], the total conservational benefit of the reserves to the Skagerrak lobster population remains unknown. Further, our receiver array did not allow for precise triangulations of the telemetric signal emitted by lobsters carrying acoustic tags. This would require that signals are picked up by at least three receivers, which was not possible given the topography and human activities (e.g., deep areas, shipping lanes, fishing) in the area. Hence, we acknowledge that some of the lobsters that we received the signal from could at times transmit from immediately outside the reserve. That being said, only one individual was lost from the telemetry study (i.e., with a ceased signal by unknown cause) during the fishing season, and this individual was not reported caught by any fisher. Moreover, most losses of lobsters from the telemetry study occurred in late summer, subsequent to the hatching season. This suggests that much of the losses were due to moulting by ovigerous females, which usually follows hatching of eggs [17]. On the other hand, moult stage [22] at start of the study did not significantly affect loss probability. Thus, some of the lost lobsters may also have died or dispersed out of the reserve. None of the eleven lobsters categorised as lost has subsequently been observed. In contrast, seven of the remaining 19 lobsters (i.e., those categorised as censored or in the study till the end) have been recaptured at least once in subsequent years of scientific fishing (until and including 2010).

### Spillover of lobsters

Skagerrak fishers recovered a low but significant portion of the T-bar tagged lobsters in the fishing grounds adjacent to the reserves (4.7%). This level of export is comparable to what has been observed for spiny lobster (Palinurus *Elephas* ) from Mediterranean marine reserves [36,37]. And since mean CPUE of lobsters has increased inside the Norwegian Skagerrak lobster reserves compared to their controls [13], which in theory corresponds to higher densities of lobsters within reserves, it is highly likely that more lobsters move out of the reserves due to density dependence [38], than the number moving into reserves against the density gradient; the very definition of the spillover effect [5]. Moreover, in concordance with a capture-mark-recapture and recovery study performed in a lobster reserve situated on the Swedish Skagerrak coast (Kåvra), where only 1.4% of 4016 lobsters tagged within the reserve were recovered 1 km or further from the reserve boundary [39], compared to 2% recovered beyond 1 km from reserve centres, the bulk spillover appears to taper off within short distances from reserves. Which is also congruent with the short movement distances observed within the reserves. Note that we have research surveys outside the reserves (in control areas), but these are limited in space and not designed to capture spillover from the reserves [13]. We recaptured none of the lobsters tagged in reserves in control areas. Furthermore, almost 53% of lobsters tagged and released within reserves were below minimum legal size at time of tagging. Since Skagerrak fishers did not ‘sample’ the sub-legal sized lobsters, the spillover reported in this study could be an underestimation. And while induced nomadism after tagging could inflate estimates of spillover, underreporting by fishers, tag loss, and a capture probability of less than 100% will deflate the magnitude, but perhaps not the extent of our estimates of spillover from reserves. That being said, we have scarce knowledge on the mobility of lobsters in fished areas. However given the relatively homogenous benthic habitat in coastal Skagerrak and the representativeness of the habitat included within the reserves, we do not expect lobsters to behave differently because of habitat differences inside compared to outside the reserves; but we do not preclude that density dependence affects movement patterns outside compared to inside reserves [38]. As a further matter, the low recovery rate of tagged lobsters outside reserves compared to the recapture rate inside is not likely to arise from lower sampling effort outside the reserves; on the contrary, fishing effort within each reserve was limited to 100 trap days per year (our scientific fishing program), whereas the fishing season outside reserves lasts for two months with intense fishing pressure during the first few weeks [14].

### Gene flow in Skagerrak

Genetic structure among geographic samples was very weak (F_ST_ < 0.0001; p=0.006), where overall significance largely was due to one locus (HGC131) being deficit of heterozygotes. The overall p-value without this locus was, however, still significant. Compared to other high gene flow species analysed with microsatellite markers in the Skagerrak, including: Atlantic cod (*Gadus morhua*) [40,41], herring ( *Clupea* *harengus* ) [42], and brown crab (Cancer *pagurus*) [43], lobster F_ST_ was at least an order of magnitude lower. Given the theoretical relationship between F_ST_ and exchange of migrants in natural populations, viz. the Island model [44], connectivity of lobsters in Skagerrak is thus likely to be high. Although a recent study on cod in Skagerrak has shown that small *F*_ST_ values may be of biological relevance [45], the estimates presented herein are still an order of magnitude smaller. The confidence interval for genetic differentiation (F_ST_) in this study also overlaps zero, and may thus prove imprecise and misleading [8]. In any case we conclude that the genetic differentiation of lobsters in Skagerrak is very low and lies close to the limit of what can be detected by our methods.

### Synthesis, management implications and future directions

Both telemetry and T-bar tagged lobsters showed high site fidelity within reserves, with occasional movement distances up to 21 km away from lobster reserves. Post-settlement movement of European lobsters (≤85 mm CL) have previously been shown to be less than 6 km [46]. Combined, these estimates give a good approximation of benthic phase movement of lobsters. Movement over longer distances (>21 km) can thus for the most part be accredited to larval drift. Palumbi [9] showed, by simulation, that isolation by distance would manifest itself beyond 2-5 times the mean larval dispersal distance, and that mean dispersal distances for fish and invertebrates with pelagic larvae probably range between 25 km and 150 km. Field studies confirm this suggestion; for example, downstream of two South African marine reserves, density of brown mussel ( *Pernaperna* ) not harvested inside the reserves decayed exponentially out to around 20 km from reserve boundaries, probably due to increased larval export form reserves into adjacent fished areas (i.e. the recruitment benefit) [47]. Also, three no-take marine reserves on the Southern Great Barrier Reef (Australia) conferred recruitment benefits to fished reefs out to a radius of 30 km in the case of two species targeted by fishers, coral trout ( *Plectropomus* *maculatus* ) and stripey snapper ( *Lutjanus* *carponatus* ) [48]. However, since Skagerrak lobsters did not show any isolation by distance within our study area, mean larval dispersal distance probably extend beyond our sampling regions, at least along parts of the coast where currents are strong. Adopting Palumbi’s [11] terminology, the lobster ‘spillover cloud’ (i.e., the extent to which animals protected inside reserves move outside reserve boundaries and then enter the local fisheries) only reaches within our sampling regions (≤21 km), whereas their ‘larval neighbourhood’ (i.e., the area centered on a set of parents that is large enough to retain most of the offspring of those parents) probably extend well beyond sampling regions, depending on variations in life conditions in the water column (e.g., currents, larval duration depending on temperature, and larval survival) [49].

To conclude, future lobster reserves could be relatively small (≥0.5 km^2^) and still protect a large portion of its inhabitants over extended time periods, and at the same time provide significant exports to adjacent fishing grounds–albeit largely on a local scale. Conversely, due to the high connectivity of lobster in Skagerrak, larvae produced within reserves could end up far beyond their borders, and even drift along the entire length of the Skagerrak coast. This study was bound by the limitations of using gene flow as proxies for small-scale larval dispersal. Further studies are thus needed on lobster larvae dispersal trajectories and retention mechanisms and patterns (see 39) to pinpoint where reserves should be placed, to maximise recruitment benefits and connectivity among reserves for this species. We also suggest comparing mobility of lobsters in fished and protected areas as an interesting future research topic

## Supporting Information

Figure S1 **CPUE of lobsters in Skagerrak.** Data on catch-per-unit-effort (CPUE) reported to the Norwegian Institute of Marine Research (IMR) from 1928 to 2012. During this period IMR have collaborated with selected fishers operating in southern and western Norway in a long standing effort to capture year to year differences in CPUE as an indicator of stock status and as a supplement to less reliable landings data [14].
(TIF)

Figure S2 **Telemetry range test.** Results from the range test performed within the Kvernskjær lobster reserve before the telemetry study. Grey circles represent the array of VR2W receivers, and the stapled line represents the reserve border. Black circles represent a position from where the signal was received by up to six receivers (i.e. circle size indicates ‘coverage’ within the reserve), and crosses represents areas from where the signal was not received.
(TIF)

Figure S3 **Size distribution of lobsters tagged and released within the reserves.** Size distribution of lobsters tagged within reserves, separated by (A) males and (B) females.
(TIF)

Figure S4 **Testing whether selection affects sampled locus.** Results from the LOSITAN analysis [30], where heterozygosity (*H*) is plotted against the *F*_ST_ for each locus. A point above the confidence envelope would indicate that directional selection was affecting a locus, whereas a point below the envelope would suggest balancing selection was affecting a locus.
(TIF)

Figure S5 **Size distribution of recovered lobsters.** Size distribution of lobsters tagged within reserves and recovered by fishers outside the reserves, separated by (A) males and (B) females.
(TIF)

Table S1 **Information on lobsters used in the telemetry study.** Information includes: group (female, male, or ovigerous female); carapace length (CL); total length (TL); moult stage at start of study (the succession of the moult stages is: C_4_, D_0_, D_1_, D_2_); number of times (N) the individual has been captured during scientific fishing programme within Kvernskjær lobster reserve; number of days the individual was present and moving within the reserve during the telemetry study; and whether individuals were lost, censored, or in the study when the study ended.
(DOCX)

Table S2 **Number of lobsters tagged and released within the reserves.** Number of lobsters tagged within Kvernskjær, Flødevigen, and Bolærne lobster reserves over five years of scientific fishing.
(DOCX)

Table S3 **Ordered loss times of lobsters in telemetry study.** A table containing the ordered loss times used to calculate the Kaplan-Meier curve. The columns contain: the number of days into the study period until a loss (Time); the number of lobsters at risk until the time of the event (N risk); number of lobsters lost at that particular day (N event); and the Kaplan-Meier survival probability to survive past the time of the previous event (Survival) with its 95% CI. *Note that the fishing season starts at 35 days into the study period, and ends 95 days into the period.
(DOCX)

Table S4 **Genetic variability within geographic samples.** Information includes: average heterozygosity (*H*_S_); allelic richness; and HW disequilibrium within each sampled site measured as *F*_IS_, along with p-values from probability tests (H_1_ = excess or deficiency of heterozygotes) and their FDR corrected p-values.
(DOCX)

Table S5 **Matrix containing Pairwise *F*_ST_.** Pairwise *F*_ST_ (below the diagonal) and p-values from allele-frequency tests (none of which were significant after FDR corrections) between Bolærne (BOL), Gullmar fjord (GUL), inner Oslofjord (IOS), Tisler (TIS), Kåvra (KVA), Singlefjord (SIN), Flødevigen (FLV), and Mandal (MAN) sampling sites.
(DOCX)
