# Supplementary figures and images for: Conservation, Spillover and Gene Flow within a Network of Northern European Marine Protected Areas
Source: PLoS One. 2013 Sep 6;8(9):e73388. doi: 10.1371/journal.pone.0073388 (PMC3765458; doi:10.1371/journal.pone.0073388)

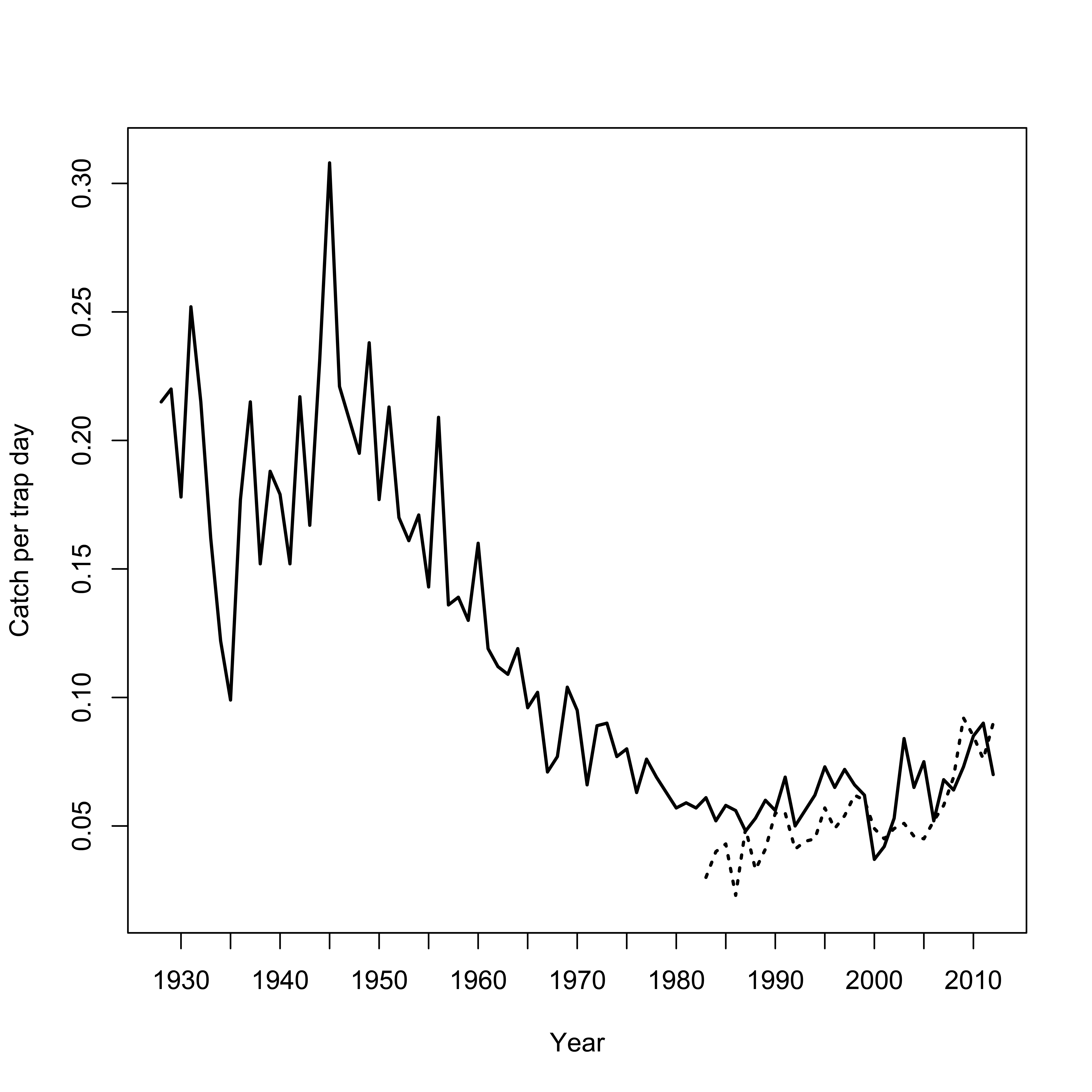

Supplement: Figure S1 — CPUE of lobsters in Skagerrak. Data on catch-per-unit-effort (CPUE) reported to the Norwegian Institute of Marine Research (IMR) from 1928 to 2012. During this period IMR have collaborated with selected fishers operating in southern and western Norway in a long standing effort to capture year to year differences in CPUE as an indicator of stock status and as a supplement to less reliable landings data [14]. (TIF) [file pone.0073388.s001.tif]

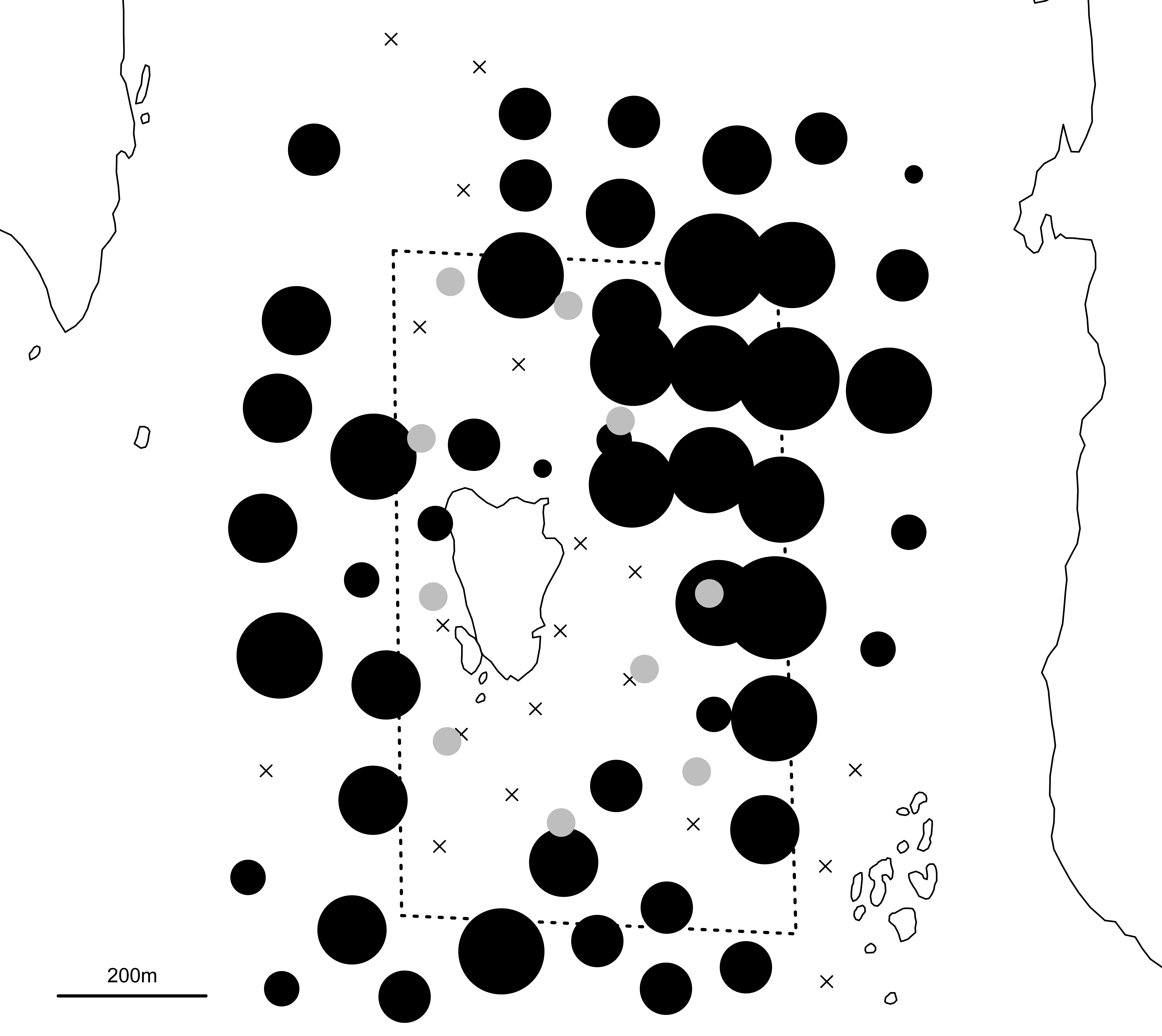

Supplement: Figure S2 — Telemetry range test. Results from the range test performed within the Kvernskjær lobster reserve before the telemetry study. Grey circles represent the array of VR2W receivers, and the stapled line represents the reserve border. Black circles represent a position from where the signal was received by up to six receivers (i.e. circle size indicates ‘coverage’ within the reserve), and crosses represents areas from where the signal was not received. (TIF) [file pone.0073388.s002.tif]

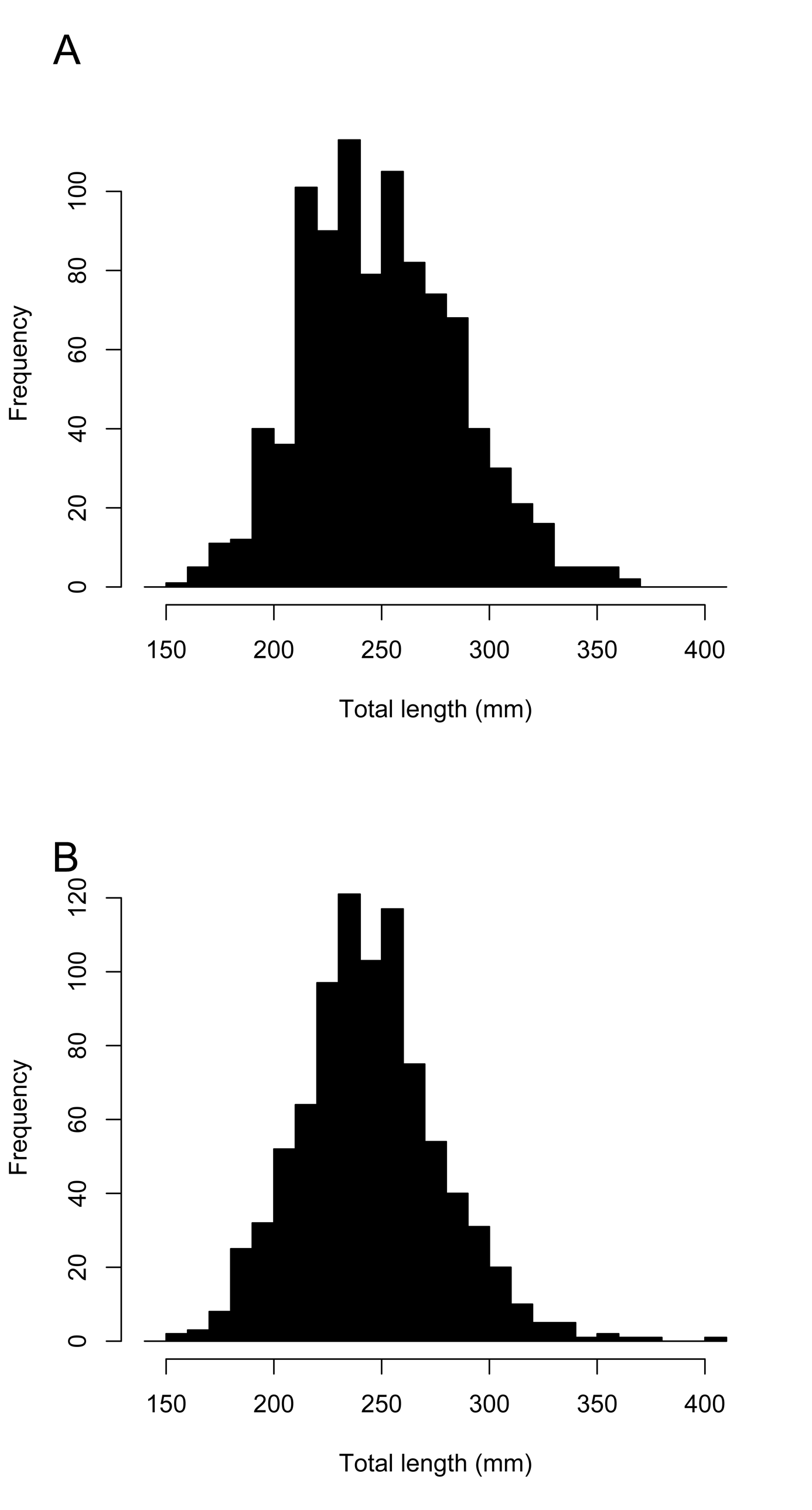

Supplement: Figure S3 — Size distribution of lobsters tagged and released within the reserves. Size distribution of lobsters tagged within reserves, separated by (A) males and (B) females. (TIF) [file pone.0073388.s003.tif]

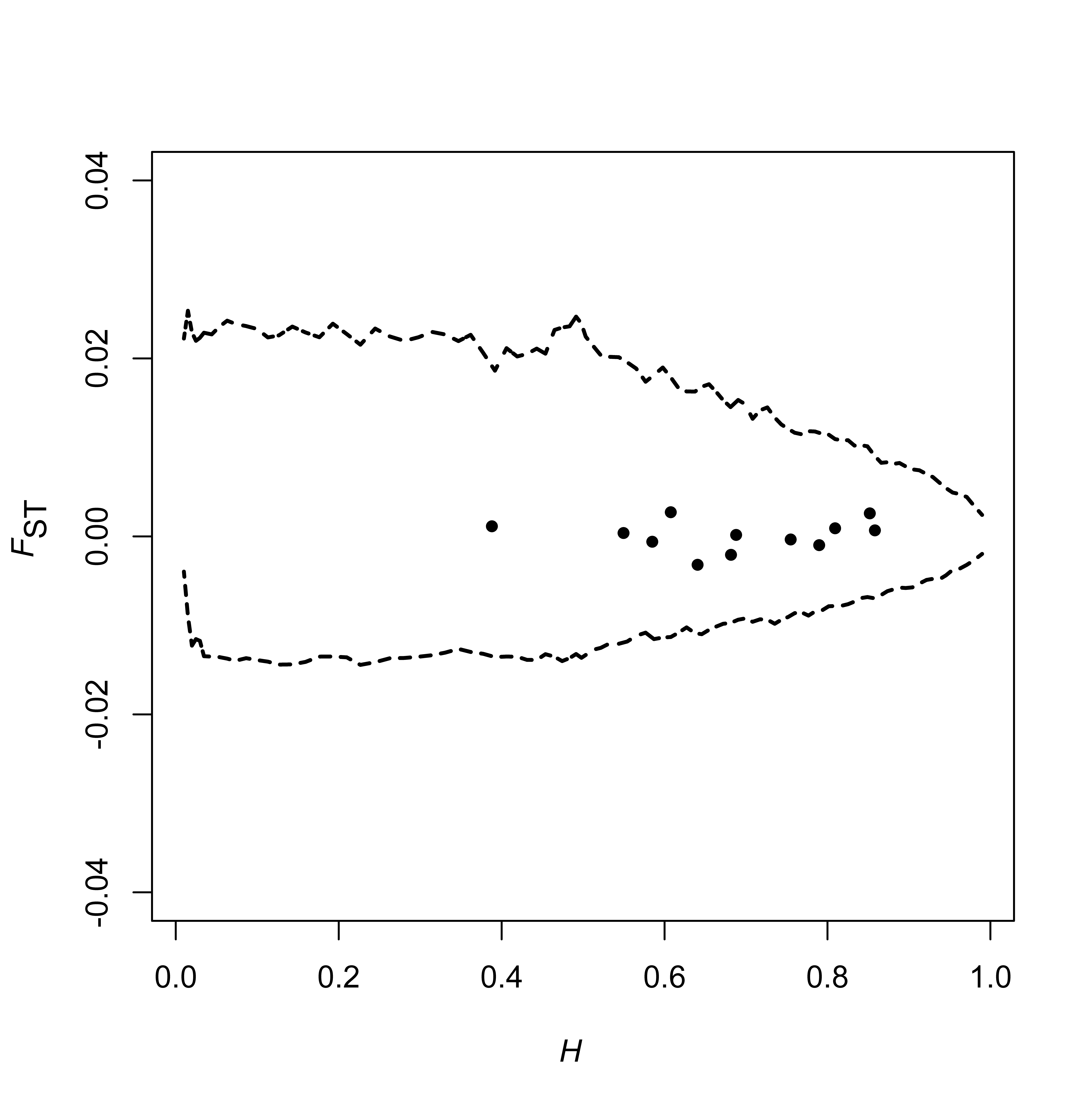

Supplement: Figure S4 — Testing whether selection affects sampled locus. Results from the LOSITAN analysis [30], where heterozygosity (H) is plotted against the F ST for each locus. A point above the confidence envelope would indicate that directional selection was affecting a locus, whereas a point below the envelope would suggest balancing selection was affecting a locus. (TIF) [file pone.0073388.s004.tif]

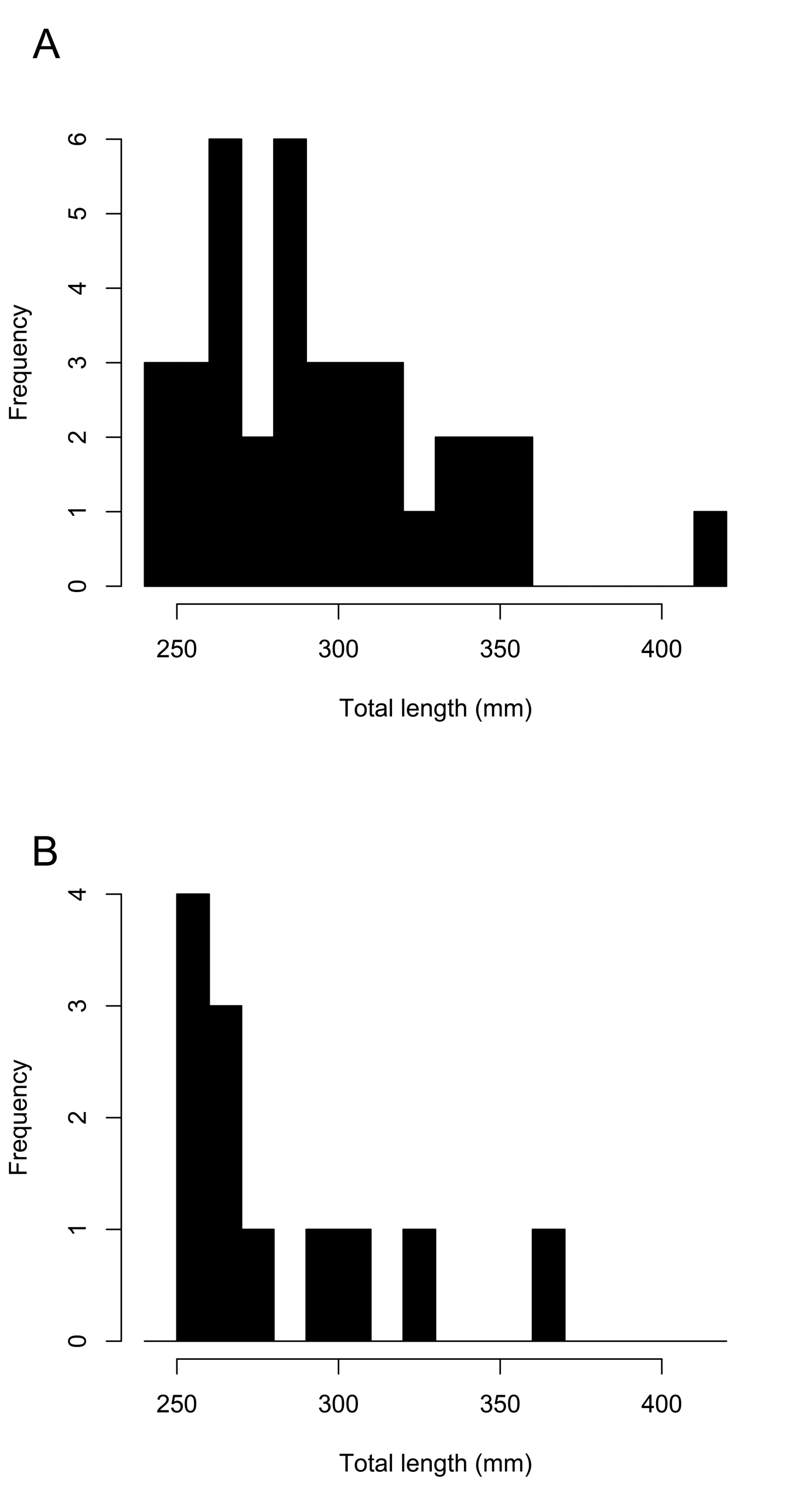

Supplement: Figure S5 — Size distribution of recovered lobsters. Size distribution of lobsters tagged within reserves and recovered by fishers outside the reserves, separated by (A) males and (B) females. (TIF) [file pone.0073388.s005.tif]
