# Supplementary material for: Conservation, Spillover and Gene Flow within a Network of Northern European Marine Protected Areas
Source: PLoS One. 2013 Sep 6;8(9):e73388. doi: 10.1371/journal.pone.0073388 (PMC3765458; doi:10.1371/journal.pone.0073388)
Supplement: Table S1 — Information on lobsters used in the telemetry study. Information includes: group (female, male, or ovigerous female); carapace length (CL); total length (TL); moult stage at start of study (the succession of the moult stages is: C4, D0, D1, D2); number of times (N) the individual has been captured during scientific fishing programme within Kvernskjær lobster reserve; number of days the individual was present and moving within the reserve during the telemetry study; and whether individuals were lost, censored, or in the study when the study ended. (DOCX) [file pone.0073388.s006.docx]

| Group | CL (mm) | TL (mm) | Moult stage | N × captured in reserve | Days | Event |
| --- | --- | --- | --- | --- | --- | --- |
| F | 76 | 220 | D_0_ | 1 | 326 | Censored |
| F | 79 | 230 | C_4_ | 1 | 274 | Censored |
| F | 85 | 247 | D_0_ | 1 | 335 | Censored |
| F | 85 | 242 | D_0_ | 2 | 364 | - |
| F | 86 | 252 | C_4_ | 1 | 9 | Lost |
| F | 89 | 255 | D_0_ | 1 | 9 | Lost |
| F | 92 | 263 | C_4_ | 3 | 323 | Censored |
| F | 96 | 278 | D_0_ | 8 | 364 | - |
| F | 105 | 301 | D_2_ | 2 | 1 | Lost |
| F | 120 | 335 | D_2_ | 1 | 225 | Lost |
| M | 84 | 239 | C_4_ | 1 | 70 | Censored |
| M | 86 | 241 | C_4_ | 1 | 68 | Lost |
| M | 86 | 245 | D_0_ | 2 | 67 | Censored |
| M | 89 | 256 | C_4_ | 2 | 335 | Lost |
| M | 92 | 257 | D_0_ | 3 | 355 | Censored |
| M | 94 | 262 | D_1_ | 2 | 30 | Censored |
| M | 98 | 280 | D_1_ | 2 | 299 | Censored |
| M | 100 | 287 | D_1_ | 1 | 20 | Censored |
| M | 112 | 315 | D_2_ | 1 | 364 | - |
| M | 122 | 342 | C_4_ | 2 | 310 | Censored |
| O | 81 | 238 | C_4_ | 3 | 364 | - |
| O | 84 | 247 | C_4_ | 3 | 93 | Censored |
| O | 84 | 247 | D_2_ | 3 | 281 | Censored |
| O | 86 | 249 | C_4_ | 2 | 364 | - |
| O | 86 | 241 | D_0_ | 3 | 353 | Censored |
| O | 88 | 250 | C_4_ | 2 | 349 | Lost |
| O | 89 | 253 | C_4_ | 2 | 363 | Lost |
| O | 89 | 250 | D_1_ | 1 | 344 | Lost |
| O | 92 | 265 | C_4_ | 2 | 343 | Lost |
| O | 98 | 273 | D_0_ | 1 | 334 | Lost |
