# Supplementary material for: Conservation, Spillover and Gene Flow within a Network of Northern European Marine Protected Areas
Source: PLoS One. 2013 Sep 6;8(9):e73388. doi: 10.1371/journal.pone.0073388 (PMC3765458; doi:10.1371/journal.pone.0073388)
Supplement: Table S2 — Number of lobsters tagged and released within the reserves. Number of lobsters tagged within Kvernskjær, Flødevigen, and Bolærne lobster reserves over five years of scientific fishing. (DOCX) [file pone.0073388.s007.docx]

| Location | 2006 | 2007 | 2008 | 2009 | 2010 | Total |
| --- | --- | --- | --- | --- | --- | --- |
| Bolærne | 67 | 146 | 106 | 246 | 187 | 752 |
| Flødevigen | 102 | 53 | 58 | 79 | 239 | 531 |
| Kvernskjær | 45 | 136 | 110 | 116 | 120 | 527 |
| Total | 214 | 335 | 274 | 441 | 546 | 1810 |
