# Supplementary material for: Conservation, Spillover and Gene Flow within a Network of Northern European Marine Protected Areas
Source: PLoS One. 2013 Sep 6;8(9):e73388. doi: 10.1371/journal.pone.0073388 (PMC3765458; doi:10.1371/journal.pone.0073388)
Supplement: Table S3 — Ordered loss times of lobsters in telemetry study. A table containing the ordered loss times used to calculate the Kaplan-Meier curve. The columns contain: the number of days into the study period until a loss (Time); the number of lobsters at risk until the time of the event (N risk); number of lobsters lost at that particular day (N event); and the Kaplan-Meier survival probability to survive past the time of the previous event (Survival) with its 95% CI. *Note that the fishing season starts at 35 days into the study period, and ends 95 days into the period. (DOCX) [file pone.0073388.s008.docx]

| Time* | N risk | N event | Survival | Lower 95%CI | Upper 95%CI |
| --- | --- | --- | --- | --- | --- |
| 1 | 30 | 1 | 0.967 | 0.905 | 1.000 |
| 9 | 29 | 2 | 0.900 | 0.799 | 1.000 |
| 68 | 24 | 1 | 0.863 | 0.746 | 0.998 |
| 225 | 21 | 1 | 0.821 | 0.690 | 0.978 |
| 334 | 14 | 1 | 0.763 | 0.608 | 0.957 |
| 335 | 13 | 1 | 0.704 | 0.534 | 0.928 |
| 343 | 11 | 1 | 0.640 | 0.459 | 0.893 |
| 344 | 10 | 1 | 0.576 | 0.389 | 0.853 |
| 349 | 9 | 1 | 0.512 | 0.325 | 0.807 |
| 363 | 6 | 1 | 0.427 | 0.239 | 0.761 |
