# Supplementary material for: Conservation, Spillover and Gene Flow within a Network of Northern European Marine Protected Areas
Source: PLoS One. 2013 Sep 6;8(9):e73388. doi: 10.1371/journal.pone.0073388 (PMC3765458; doi:10.1371/journal.pone.0073388)
Supplement: Table S4 — Genetic variability within geographic samples. Information includes: average heterozygosity (H S); allelic richness; and HW disequilibrium within each sampled site measured as F IS, along with p-values from probability tests (H1 = excess or deficiency of heterozygotes) and their FDR corrected p-values. (DOCX) [file pone.0073388.s009.docx]

|  |  |  | HW disequilibrium | | |
| --- | --- | --- | --- | --- | --- |
| Sample site | *H*_S_ | Allelic richness | *F*_IS_ | P-values | FDR corr. |
| Gullmar fjord | 0.698 | 8.101 | -0.009 | 0.288 | 0.384 |
| Kåvra | 0.669 | 8.218 | 0.024 | <0.001 | <0.001 |
| Tisler | 0.650 | 8.152 | 0.041 | 0.027 | 0.055 |
| Singlefjord | 0.664 | 8.008 | 0.021 | 0.268 | 0.384 |
| Inner Oslofjord | 0.664 | 8.528 | 0.034 | <0.001 | <0.001 |
| Bolærne | 0.674 | 8.129 | 0.007 | 0.768 | 0.791 |
| Flødevigen | 0.673 | 8.146 | 0.011 | 0.790 | 0.791 |
| Mandal | 0.661 | 8.180 | 0.037 | 0.004 | 0.011 |
