# Supplementary material for: Conservation, Spillover and Gene Flow within a Network of Northern European Marine Protected Areas
Source: PLoS One. 2013 Sep 6;8(9):e73388. doi: 10.1371/journal.pone.0073388 (PMC3765458; doi:10.1371/journal.pone.0073388)
Supplement: Table S5 — Matrix containing Pairwise FST. Pairwise F ST (below the diagonal) and p-values from allele-frequency tests (none of which were significant after FDR corrections) between Bolærne (BOL), Gullmar fjord (GUL), inner Oslofjord (IOS), Tisler (TIS), Kåvra (KVA), Singlefjord (SIN), Flødevigen (FLV), and Mandal (MAN) sampling sites. (DOCX) [file pone.0073388.s010.docx]

|  | BOL | GUL | IOS | TIS | KVA | SIN | FLV | MAN |
| --- | --- | --- | --- | --- | --- | --- | --- | --- |
| BOL | - | 0.350 | 0.276 | 0.048 | 0.299 | 0.020 | 0.019 | 0.266 |
| GUL | 0.0005 | - | 0.213 | 0.603 | 0.253 | 0.071 | 0.140 | 0.601 |
| IOS | -0.0017 | 0.0000 | - | 0.041 | 0.347 | 0.392 | 0.037 | 0.172 |
| TIS | 0.0018 | 0.0014 | -0.0007 | - | 0.146 | 0.668 | 0.120 | 0.405 |
| KVA | -0.0017 | -0.0005 | -0.0017 | 0.0001 | - | 0.126 | 0.170 | 0.092 |
| SIN | 0.0020 | 0.0017 | -0.0003 | -0.0003 | -0.0001 | - | 0.013 | 0.084 |
| FLV | 0.0012 | 0.0003 | -0.0012 | 0.0001 | 0.0000 | 0.0011 | - | 0.461 |
| MAN | 0.0015 | -0.0008 | -0.0007 | -0.0007 | -0.0014 | 0.0011 | -0.0011 | - |
